# Impact of the Female Genital Microbiota on Outcomes of Assisted Reproductive Techniques

**DOI:** 10.3390/biomedicines13061332

**Published:** 2025-05-29

**Authors:** Zacharias Fasoulakis, Dimitrios Papageorgiou, Athanasios Papanikolaou, Marianna Chatziioannou, Ioakeim Sapantzoglou, Afroditi Pegkou, George Daskalakis, Panos Antsaklis

**Affiliations:** 1 1st Department of Obstetrics and Gynecology, National and Kapodistrian University of Athens, General Hospital Alexandra, 15238 Athens, Greece; 2 Athens Naval and Veterans Hospital, 11521 Athens, Greece

**Keywords:** genital microbiota, vaginal microbiota, endometrial microbiota, bacteria, assisted reproductive techniques, ART, fertility

## Abstract

The female genital microbiota plays a critical role in reproductive health and has recently emerged as a key factor influencing the outcomes of Assisted Reproductive Techniques (ARTs). Beyond traditional concerns about vaginal dysbiosis and infections such as bacterial vaginosis or mycoses, recent evidence highlights the broader impact of genital microbial communities, including the vaginal, cervical, and endometrial niches, on ART success rates. New findings suggest that specific bacterial profiles, as well as shifts in the virome and mycobiome, can significantly affect implantation and pregnancy outcomes. Non-invasive biomarkers such as menstrual blood have also been proposed for assessing endometrial receptivity. Furthermore, growing attention has been directed towards methodological challenges such as contamination risks during microbiota sampling which may influence study reliability. This review synthesizes the latest data on the relationship between the female genital microbiota and ART outcomes, with a focus on standardized microbiological analysis techniques and specific patient populations such as those experiencing recurrent implantation to optimize ART success based on microbiota profiling.

## 1. Introduction

Microorganisms are ubiquitous in the female genital tract [1,2,3,4], playing integral roles in maintaining reproductive health, influencing fertility, and protecting against pathogens. However, the diversity of the genital microbiota is generally low compared to other microbiota communities found within the human body, such as those in the gastrointestinal or oral regions [5,6,7,8,9]. This lower diversity is particularly evident in the vaginal microbiota, which is distinctively different from other mammalian species in terms of microbial composition [10,11,12,13,14,15].

Unlike many other mammals, the human vaginal microbiota is predominantly colonized by a single bacterial genus: Lactobacillus. These lactobacilli play a vital role in maintaining a healthy vaginal ecosystem by producing lactic acid, which creates an acidic environment (pH around 4.5) unfavorable to pathogenic microorganisms [13,14,15]. Additionally, lactobacilli produce antimicrobial compounds, such as hydrogen peroxide (H_2_O_2_), bacteriocins, and biosurfactants, further enhancing their protective function against opportunistic pathogens [13].

The female genital microbiota exhibits distinct variations between the lower and upper genital tracts. The microbiota of the lower genital tract, including the vagina, is highly abundant in bacterial cells but demonstrates relatively low microbial diversity, dominated primarily by lactobacilli [10,11,12,13,14]. Conversely, the upper genital tract, comprising the endometrial cavity, fallopian tubes, and ovaries, harbors a microbiota that is considerably less abundant but more diverse in terms of species richness [10,11,12,13,14]. The stark contrast between the microbial composition and diversity of the upper and lower genital tracts suggests that physiological and immunological mechanisms are at play, limiting bacterial migration and colonization of the upper reproductive regions.

One prevalent theory explaining this microbial gradient involves the anatomical and physiological role of the cervix, acting as a physical barrier that restricts the ascension of microorganisms from the vagina into the uterine cavity [12]. The cervix’s mucus plug and immunological defenses are proposed to selectively prevent bacterial migration, ensuring minimal contamination of the uterus and fallopian tubes. Another complementary hypothesis attributes the lower bacterial abundance in the upper genital tract to the enhanced immune surveillance and more robust immunological responses within the endometrial lining, which effectively neutralize and eliminate microbial invaders that do manage to traverse the cervical barrier [12].

Notably, although Lactobacillus species dominate the vaginal, endometrial, and tubal microbiota, emerging evidence suggests the cervical microbiota may possess a distinct microbial signature, with a noticeable prevalence of bacteria from the Mycoplasmataceae family [11,13,15]. Mycoplasmataceae, which include organisms such as Mycoplasma and Ureaplasma, have been implicated in reproductive tract infections and adverse reproductive outcomes, emphasizing the clinical importance of understanding the unique microbial niches within the genital tract. These regional microbiota differences underscore the complexity of microbial ecosystems and their importance for reproductive health.

Although traditionally studied in the context of infections and dysbiosis, recent evidence suggests that the female genital microbiota plays a much broader role in shaping the outcomes of Assisted Reproductive Techniques (ARTs), including in vitro fertilization (IVF) and embryo transfer. ART procedures are highly sensitive to the microbial environment of the reproductive tract, with the composition of vaginal, cervical, and endometrial microbiota potentially influencing implantation success, endometrial receptivity, and pregnancy rates through mechanisms such as immune modulation and inflammatory signaling. While growing evidence supports these associations, the underlying mechanisms remain incompletely understood, and clinical application is still limited by methodological heterogeneity. This review synthesizes the most recent findings on how distinct microbial communities across different compartments of the female reproductive tract affect ART outcomes. Special emphasis is placed on methodological challenges such as contamination risk, the value of non-invasive biological substrates like menstrual blood, and the clinical importance of microbiota profiling in specific populations—particularly women experiencing recurrent implantation failure (RIF). By consolidating current knowledge and highlighting potential diagnostic and therapeutic strategies, this review aims to support the integration of microbial assessment into personalized ART protocols.

## 2. Genital Microbiota

Microorganisms are ubiquitously present throughout the female genital tract [8,10,11,12]. However, the diversity of the genital microbiota is generally low compared to other microbiota in the human body [12]. Unlike other mammals, the human vaginal microbiota has a dominant bacterial species: lactobacilli [13,14,15]. The microbiota of the lower genital tract is very abundant yet not very diverse, whereas the microbiota of the upper genital tract is less abundant but more diverse [10,11,12,13,14]. This suggests that the cervix may act as a barrier, preventing the upward movement of microorganisms from the vagina to the uterus [12]. Another hypothesis is that the endometrial immune response more effectively destroys microorganisms than that of the vagina [12]. Bacteria of the Lactobacilaceae family, particularly Lactobacillus or lactobacilli, constitute the majority of microorganisms in the vaginal, endometrial, and tubal microbiota [11,13]. However, bacteria of the Mycoplasmataceae family appear to predominate in the cervical microbiota [11,13,15].

### 2.1. Vaginal Microbiota

The concentration of lactobacilli is between 10^7^ and 10^9^ colony-forming units (CFUs) per gram of vaginal secretion. Other bacteria form a minority of the vaginal microbiota, such as those from the Bifidobacteriaceae, Coriobacteriaceae, Enterobacteriaceae, and Streptococcaceae families [13]. The vaginal microbiota thus demonstrates lower alpha diversity (the number of species coexisting in a given environment) as well as lower beta diversity (the comparison of species diversity between different environments) compared to other human body sites [2,16,17].

#### 2.1.1. Influence of Ethnicity and Geography on Vaginal Microbiota

Recent studies have revealed significant variations in vaginal microbiota composition associated with ethnicity and geographic regions. Ravel et al. demonstrated that African American and Hispanic women predominantly exhibited Community State Type (CST) IV, characterized by increased microbial diversity and a reduced abundance of lactobacilli compared to Caucasian and Asian women, who primarily displayed Lactobacillus-dominated CSTs, especially CST I, dominated by Lactobacillus crispatus [18]. Geographical differences have also been noted; women from Sub-Saharan Africa and South Asia frequently show vaginal microbiota profiles with higher diversity and lower lactobacilli abundance, potentially due to genetic, environmental, or socio-cultural factors influencing microbial composition and reproductive health outcomes [19,20]. These ethnic and geographical variations underline the necessity for personalized approaches to diagnosing and treating genital dysbiosis.

#### 2.1.2. Classification of the Vaginal Microbiota

The vaginal microbiota is commonly classified into five Community State Types (CSTs) among women of childbearing age, four of which are dominated by lactobacilli: Lactobacillus crispatus (CST I), Lactobacillus gasseri (CST II), Lactobacillus iners (CST III), and Lactobacillus jensenii (CST V) [18]. Unlike the aforementioned CSTs, lactobacilli are in the minority in CST IV, which is primarily composed of anaerobic bacteria (notably Gardnerella, Prevotella, Corynebacterium, Sneathia, Mobiluncus, Atopobium, and Megasphaera) [18]. The distribution of the five CSTs is not uniform among women of childbearing age [13,18,19,20].

#### 2.1.3. Impact of Lifestyle Factors

Lifestyle factors, including diet, hygiene practices, and sexual behaviors, significantly influence vaginal microbiota composition. Dietary patterns, particularly those high in sugars or refined carbohydrates, have been associated with an increased prevalence of vaginal candidiasis due to the promotion of Candida growth [21,22]. Additionally, frequent vaginal douching and the use of hygiene products containing antiseptics have been linked to reduced lactobacilli populations, contributing to increased microbial diversity and vulnerability to infections such as bacterial vaginosis [23]. Sexual behaviors, including the frequency and type of sexual activity, condom use, and number of sexual partners, also substantially affect microbiota stability, potentially increasing exposure to pathogens and altering the balance of protective microorganisms [24]. Therefore, understanding and modifying lifestyle factors could represent a crucial element in managing vaginal microbiota health.

#### 2.1.4. Functions and Common Variations of the Vaginal Microbiota

The vaginal microbiota serves a protective role against certain pathogens [25]. Estrogens and progesterone stimulate glycogen release by the vaginal epithelium, which lactobacilli metabolize into lactic acid, keeping the vaginal pH at around 4.5 [26,27]. Lactobacilli also produce hydrogen peroxide (H_2_O_2_) to eradicate specific anaerobic microorganisms [28].

The vaginal microbiota demonstrates intra- and inter-individual variability influenced by physiological parameters such as age, ethnicity, menstrual cycle, ovarian stimulation, pregnancy, and sexual activity [18,19,20,25,26,27,28]. This variability complicates the distinction between physiological and pathological microbiota. For instance, the microbiota associated with asymptomatic bacterial vaginosis—which constitutes 50% of bacterial vaginosis cases [29]—overlaps with CST IV of Ravel’s classification, challenging the pathogenicity of asymptomatic bacterial vaginosis and the reliability of dysbiosis diagnostic criteria [30,31,32].

Dysbiosis with symptoms like leukorrhea, pruritus, or vulvodynia requires treatment. Bacterial vaginosis occurs two to four times more frequently in infertile patients, suggesting that vaginal dysbiosis could negatively affect spontaneous female fertility [33,34,35]. Further research is necessary to define “pathological” vaginal microbiota and identify dysbiosis that endangers women’s health.

### 2.2. Endometrial Microbiota

Research into the endometrial microbiota is more recent than that of the vaginal microbiota. One of the major challenges in studying the endometrial microbiota is avoiding contamination from the lower genital tract during sample collection. This contamination, often referred to as the “splashome” phenomenon, occurs when vaginal or cervical microorganisms ascend and inadvertently contaminate the endometrial sample during transcervical procedures. Such contamination can falsely suggest the presence of bacteria in the uterus, leading to misinterpretation of the microbiota composition and its potential role in reproductive outcomes. Several strategies have been developed to minimize splashome-related contamination, including the use of specialized double-lumen catheters and strict aseptic techniques during sampling [36,37]. Nevertheless, no method is entirely free from risk. Some studies now advocate for careful parallel sampling of vaginal, cervical, and endometrial microbiota, combined with high-sensitivity molecular techniques, to distinguish true endometrial microorganisms from contaminants [38]. Addressing the splashome phenomenon is essential for improving the reliability of endometrial microbiota studies and developing accurate clinical interventions based on microbial profiling. However, minimized-contamination-risk procedures have confirmed the presence of an intrinsic endometrial microbiota. It is now believed that colonization of the endometrium likely occurs via ascending migration from the vagina or hematogenously [36,39,40].

Recent metagenomic analyses indicate that the endometrial microbiota is primarily dominated by *Lactobacillus* spp., particularly *Lactobacillus crispatus*. However, other genera frequently detected in healthy endometrial microbiota include Gardnerella, Prevotella, Atopobium, Bifidobacterium, and Streptococcus. Importantly, certain bacteria typically associated with dysbiosis or reproductive pathologies, such as *Gardnerella vaginalis*, *Enterococcus faecalis*, *Escherichia coli*, and *Ureaplasma* spp., have also been identified. The presence of these potentially pathogenic or opportunistic species may lead to local inflammatory responses, endometrial epithelial disruption, and impaired embryo implantation, thus negatively influencing fertility outcomes [12,41,42]. In particular, patients with RIF have frequently been found to exhibit altered endometrial microbiota profiles, with decreased Lactobacillus dominance and increased abundance of anaerobic or pathogenic taxa such as Gardnerella, Streptococcus, and Prevotella. These dysbiotic patterns are often associated with chronic endometritis, a condition that is underdiagnosed yet significantly linked to implantation failure and reduced ART success rates [41,42,43]. Metagenomic analysis of endometrial fluid reveals a predominance of lactobacilli in most women. The endometrial microbiota’s composition appears stable across the menstrual cycle [37,44]. Recent data report microbiota stability in 81.8% of women through the prereceptive and receptive phases [37].

Additionally, menstrual blood, which is derived directly from the endometrial lining, may provide insights into the composition of the endometrial microbiota. In particular, with the use of real-time PCR analysis, Jain et al. confirmed that menstrual blood contains microbial communities reflective of the endometrial environment, primarily dominated by *Lactobacillus* spp., with the occasional presence of anaerobic or potentially pathogenic bacteria such as *Gardnerella vaginalis*, *Prevotella* spp., and *Atopobium* spp. [45]. These findings suggest that menstrual blood could serve as a potentially valuable, non-invasive source for assessing microbial composition related to endometrial receptivity and assisted reproductive outcomes, although further research is needed to validate and standardize this approach.

Although evidence linking the endometrial microbiota to implantation success is emerging and increasingly compelling, the precise mechanisms through which specific microbiota compositions influence female fertility remain incompletely understood. Certain imbalances in endometrial microbiota are associated with reproductive pathologies, including endometriosis and chronic endometritis, and dysbiosis has been associated with impaired embryo implantation likely mediated by local inflammatory responses. However, more robust studies and standardized methodologies are still required to definitively clarify these relationships [12,41,42].

### 2.3. Cervical Microbiota

Recent research has highlighted the significant role of cervical microbiota composition in influencing embryo implantation outcomes in women undergoing ARTs. A 2025 study by Wu et al. investigated the impact of lower genital tract microbiota on IVF and frozen embryo transfer (FET) outcomes. The study analyzed cervical microbiota samples from 131 women using 16S rDNA full-length sequencing and found that certain genera, notably Halomonas and Veillonella, were significantly more abundant in patients who did not achieve clinical pregnancy compared to those who did. These findings led to the development of a predictive model for embryo implantation failure, incorporating the presence of these genera as adverse factors, which demonstrated good predictive performance upon validation [17]. Complementing these findings, Bednarska et al. evaluated the cervical and endometrial bacterial microbiome in 177 women treated for infertility. The study observed that while Lactobacillus species were predominant in successful embryo transfers, the presence of other bacteria such as Escherichia coli and Gardnerella vaginalis negatively impacted the protective effect of Lactobacilli, potentially affecting implantation success [46].

These studies underscore the importance of cervical microbiota profiling in predicting ART success and suggest that modulation of the cervical microbiome could be a potential strategy to improve implantation outcomes.

## 3. Menstrual Blood as a Non-Invasive Biological Substrate for Biomarker Assessment of Endometrial Receptivity

Recent advancements in reproductive medicine suggest menstrual blood may serve as a non-invasive biomarker of endometrial receptivity, a key factor in ART success. Traditional methods for evaluating endometrial receptivity often involve invasive procedures like endometrial biopsies, which can be uncomfortable and carry certain risks. In contrast, menstrual blood offers a less invasive alternative, providing valuable insights into the endometrial environment.

Jain et al. investigated the immunological and microbiological profiles of menstrual blood in women undergoing IVF. The study analyzed samples from 42 patients, assessing 48 immune mediators using multiplex immunoassays and identifying microbiota components through real-time PCR. The findings revealed significant differences in cytokine levels, such as granulocyte colony-stimulating factor (G-CSF), interleukin-6 (IL-6), and monocyte chemoattractant protein-1 (MCP-1), between patients who achieved pregnancy and those who did not. These results suggested that specific cytokine profiles in menstrual blood may serve as predictive markers for endometrial receptivity and subsequent ART outcomes [45]. Complementing these findings, Whitbread et al. demonstrated the feasibility of using menstrual blood to estimate vitamin A and D levels, which are known to play key roles in reproductive health. The study found significant correlations between vitamin levels in menstrual and capillary blood samples, indicating that menstrual blood could be a reliable medium for monitoring nutritional status relevant to fertility [47]. Furthermore, recent data highlight the potential of menstrual-blood-derived mesenchymal stem cells (MenSCs) in regenerative medicine, emphasizing their role in endometrial repair and function. These cells exhibit properties similar to bone-marrow-derived stem cells and have shown promise in enhancing endometrial receptivity, thereby improving implantation rates in ART procedures [48].

Collectively, these studies highlight the utility of menstrual blood as a non-invasive, informative biomarker for assessing endometrial receptivity. Incorporating menstrual blood analysis into clinical practice could enhance the personalization of ART protocols, leading to improved pregnancy outcomes for patients undergoing fertility treatments.

## 4. Virome and Mycobiome in Reproductive Health

Recent advancements in reproductive microbiology have highlighted the significant roles of the virome and mycobiome—communities of viruses and fungi, respectively—in influencing female reproductive health and outcomes of ARTs. Traditionally, research has focused predominantly on bacterial components of the genital microbiota; however, emerging evidence suggests that viral and fungal constituents also play crucial roles in maintaining reproductive tract homeostasis and may impact fertility [49].

The vaginal virome comprises a diverse array of viruses, including bacteriophages and eukaryotic viruses, which interact intricately with the host and bacterial microbiota. Recent reports characterized the vaginal DNA virome in women undergoing IVF and found that alterations in the virome composition were associated with bacterial vaginosis (BV), a condition linked to adverse reproductive outcomes. Specifically, the presence of certain bacteriophages correlated with shifts in bacterial communities, suggesting that the virome may influence bacterial balance and, consequently, reproductive health [6,7]. In addition to bacteriophages, eukaryotic viruses such as human papillomavirus (HPV) have been implicated in reproductive tract pathologies. Eskew et al. explored the association between the eukaryotic vaginal virome and reproductive outcomes in women undergoing IVF. The research indicated that certain viral profiles were linked to reduced clinical pregnancy rates, underscoring the potential impact of the virome on ART success [50].

The mycobiome, though less studied, is another critical component of the genital microbiota. Fungal species, particularly *Candida* spp., are common inhabitants of the vaginal ecosystem. While often asymptomatic, overgrowth can lead to vulvovaginal candidiasis, which has been associated with inflammation and potential complications in fertility treatments. Further research is needed to elucidate the specific roles of various fungal species in reproductive health and their interactions with other microbial communities [49,51].

## 5. Impact of Female Genital Microbiota on ART Outcomes

The effect of bacterial vaginosis (BV) and broader genital dysbiosis on ART outcomes remains an active area of research. Meta-analyses have provided conflicting evidence regarding the impact of BV on pregnancy and miscarriage rates, highlighting significant methodological heterogeneity between studies [6,7]. A high-diversity vaginal microbiota, characterized by a reduced abundance of Lactobacilli and the overrepresentation of anaerobic bacteria such as Gardnerella and Prevotella, an “unfavorable vaginal microbiota” profile, has been linked to poorer ART outcomes with lower pregnancy and live birth rates in women undergoing ARTs [11].

In parallel, cervical microbiota has emerged as an important but underexplored factor in ART success. Recent studies have shown that particular bacteria in the cervical microbiota, such as Halomonas and Veillonella, are associated with increased risks of embryo implantation failure. Wu et al. proposed a predictive model for ART outcomes based on cervical microbiota profiling, demonstrating its potential utility in early risk assessment [17]. Similarly, Bednarska-Czerwińska et al. reported that while Lactobacillus dominance favored successful embryo implantation, the presence of pathogens like Escherichia coli and Gardnerella vaginalis could counteract these benefits [46].

Recent investigations into menstrual blood as a diagnostic tool have opened new perspectives for non-invasive assessment of endometrial receptivity. A pilot study demonstrated that cytokine profiles in menstrual blood, particularly granulocyte colony-stimulating factor (G-CSF) and interleukin-6 (IL-6) levels, differed significantly between women who achieved pregnancy and those who did not, suggesting its value as a biomarker for ART success [45]. Other studies also highlighted menstrual blood’s potential in assessing nutritional status (vitamins A and D) important for reproductive health [47], and the regenerative capabilities of menstrual-blood-derived mesenchymal stromal cells in enhancing endometrial receptivity [52].

Beyond bacterial communities, the vaginal virome and mycobiome have begun to attract attention in reproductive health. The vaginal virome, comprising bacteriophages and eukaryotic viruses, has been associated with bacterial community shifts and ART outcomes [51]. For instance, certain bacteriophages were found to correlate with bacterial vaginosis, while the presence of eukaryotic viruses like HPV was associated with reduced clinical pregnancy rates during IVF [50]. The vaginal mycobiome, primarily composed of Candida species, may also influence inflammatory responses detrimental to fertility, although more research is needed to elucidate its exact role [53].

The endometrial microbiota, in particular, appears to play a crucial role in implantation success. Several studies have demonstrated that an endometrium dominated by Lactobacillus species, especially Lactobacillus crispatus, is associated with higher implantation and live birth rates in IVF/ICSI cycles [54]. In contrast, endometrial dysbiosis, marked by increased microbial diversity and the presence of opportunistic pathogens such as Gardnerella, Enterococcus, and Pseudomonas, has been linked to poorer reproductive outcomes, particularly in patients with chronic endometritis or RIF [43,55]. These findings suggest that the evaluation and modulation of endometrial microbiota could emerge as a therapeutic strategy to improve ART outcomes. The clinical relevance of these findings is especially pronounced in patients with RIF, where endometrial dysbiosis and chronic endometritis appear to play key roles. Studies suggest that modulating the endometrial microbiota through targeted antibiotic or probiotic therapy may improve reproductive outcomes in this challenging patient group [43,55,56].

Overall, a growing body of evidence indicates that the composition of both the lower and upper genital tract microbiota significantly influences ART success (Table 1). Future fertility treatments are expected to increasingly integrate microbiota profiling and targeted strategies such as the use of probiotics, antibiotics, or microbiota transplantation to enhance ART outcomes and improve patient care.

### 5.1. Clinical Evidence on Endometrial Microbiota and ART

Recent studies have strengthened the evidence linking endometrial microbiota composition to ART outcomes. Moreno et al. (2022) demonstrated that an endometrial microbiota dominated by Lactobacillus (>90%) was associated with significantly higher implantation rates and live births compared to microbiota profiles with increased bacterial diversity and anaerobic dominance, such as Gardnerella and Atopobium [57]. Similarly, Lozano et al. (2023) reported that endometrial dysbiosis, characterized by increased abundance of opportunistic pathogens like Prevotella and Streptococcus, was frequently observed in patients with RIF, underscoring the potential role of microbiota in impaired endometrial receptivity [56]. However, methodological variations, such as differences in sampling techniques, sequencing depth, and approaches to mitigate contamination, limit the comparability and generalizability of these findings, necessitating standardized protocols to enhance future clinical applicability.

#### 5.1.1. Mechanistic Insights

The mechanisms through which the endometrial microbiota influences reproductive outcomes primarily involve modulation of local immune responses and inflammatory pathways. An imbalanced endometrial microbiota can provoke an altered cytokine environment, characterized by elevated pro-inflammatory cytokines (e.g., IL-1β, IL-6, TNF-α) and reduced anti-inflammatory mediators, potentially impairing embryo implantation by creating a hostile uterine environment [9]. Additionally, pathogenic microbes may disrupt endometrial epithelial integrity and stimulate Toll-like receptor (TLR)-mediated immune activation, further exacerbating local inflammation and diminishing endometrial receptivity [58]. These insights highlight the complex interaction between microbiota and host immune responses, reinforcing the importance of microbial homeostasis for successful implantation.

#### 5.1.2. Diagnostic and Therapeutic Potential

Advanced molecular techniques, particularly next-generation sequencing (NGS) and quantitative PCR, have significantly improved the accuracy and resolution of endometrial microbiota profiling in clinical practice, facilitating the identification of specific microbial signatures linked to fertility outcomes [8]. Clinically, these methodologies enable targeted therapeutic interventions, including the selective use of antibiotics to reduce pathogenic bacteria or probiotics aimed at restoring beneficial microbial populations, particularly Lactobacilli [10]. Preliminary trials involving probiotic supplementation, such as intravaginal administration of Lactobacillus crispatus, have shown promising results in improving endometrial microbiota composition, thereby potentially enhancing ART success rates [15]. However, further large-scale, randomized controlled trials are needed to confirm the efficacy, safety, and long-term impacts of microbiota-targeted therapies in reproductive medicine.

## 6. Conclusions

The intricate relationship between the female genital microbiota and reproductive success has become increasingly evident in recent research. Historically, investigations primarily focused on vaginal dysbiosis and its well-documented adverse impacts on ART outcomes. However, contemporary advancements have significantly expanded this perspective, revealing critical insights into the roles of previously overlooked microbial niches, such as the cervical and endometrial microbiota, as well as emerging biomarkers detectable in menstrual blood. Additionally, there is growing recognition of the clinical relevance of non-bacterial communities, including the virome and mycobiome, emphasizing the complexity of interactions within the reproductive microbiological environment.

Accumulating evidence consistently supports the notion that a Lactobacillus-dominated microbiota, both vaginally and endometrially, provides a beneficial environment for successful implantation and live birth. In contrast, dysbiotic conditions characterized by reduced lactobacilli populations or overgrowth of anaerobic and pathogenic microorganisms such as Gardnerella, Prevotella, or Escherichia coli are strongly associated with decreased fertility outcomes, including higher rates of implantation failure and pregnancy loss. These findings underline the importance of microbiota homeostasis in reproductive health and the necessity of identifying strategies for microbiota modulation to optimize fertility treatment success.

Recent advances in diagnostic approaches further emphasize the clinical utility of microbiota characterization in ARTs. Cervical microbiota profiling, enabled by sophisticated molecular sequencing technologies, has emerged as a promising tool to predict implantation outcomes and individualize patient management strategies. Similarly, non-invasive biomarkers in menstrual blood, including cytokines, vitamins, and mesenchymal stromal cells, offer innovative approaches for assessing endometrial receptivity without the discomfort and risk associated with traditional invasive methods. Despite these promising developments, methodological challenges, particularly the “splashome” phenomenon—contamination during endometrial sampling from the lower genital tract—continue to pose significant hurdles. Addressing these technical limitations remains crucial for obtaining reliable and clinically relevant microbiota profiles.

Nevertheless, the translation of microbiota profiling from research settings into routine clinical practice is still limited by considerable methodological heterogeneity across studies, a lack of standardized protocols for sampling and analysis, and the need for validation through larger prospective trials. Future research directions should prioritize standardizing procedures to enhance comparability between studies, validating microbiota-based predictive models in diverse populations, and exploring therapeutic interventions targeting microbiota modulation. Interventions such as probiotics, targeted antibiotics, and possibly microbiota transplantation represent exciting therapeutic possibilities that require thorough evaluation regarding efficacy, safety, and long-term reproductive outcomes.

Ultimately, integrating microbiota assessment into personalized reproductive medicine offers substantial potential to improve ART success rates. By tailoring treatment strategies based on individualized microbiota profiles, clinicians can enhance endometrial receptivity, reduce inflammatory disturbances, and thereby optimize fertility outcomes. In clinical contexts such as RIF, where conventional causes may be absent, microbial profiling—particularly of the endometrium—offers promising diagnostic and therapeutic opportunities. Continued advancement in this dynamic area of research promises not only to enrich our fundamental understanding of reproductive biology but also to significantly elevate standards of patient care within ART programs.

## Figures and Tables

**Table 1:** Overview of microbiota niches and their impact on ART outcomes.

Microbiota Niche	Main Characteristics	Key Findings Related to ART	References
**Vaginal Microbiota**	Dominated by Lactobacillus species; low diversity	Dysbiosis associated with lower pregnancy and live birth rates	[6,7,11]
**Cervical Microbiota**	Higher prevalence of Halomonas, Veillonella, and Mycoplasmataceae in dysbiosis	Certain bacterial profiles (e.g., Halomonas) linked to implantation failure	[17,46]
**Endometrial Microbiota**	Lower bacterial load; mostly Lactobacillus crispatus dominance	Dysbiosis associated with recurrent implantation failure (RIF) and chronic endometritis	[40,43,54,55]
**Virome**	Presence of bacteriophages and eukaryotic viruses (e.g., HPV)	Alterations associated with bacterial vaginosis and reduced ART success	[49,50,51]
**Mycobiome**	Mainly Candida spp.	Possible association with inflammation and implantation failure; role under investigation	[49,51]

## Abbreviations

The following abbreviations are used in this manuscript:

IVF	in vitro fertilization
ART	Assisted Reproductive Techniques
CFU	colony-forming units
CST	Community State Types
COS	Controlled Ovarian Stimulation
RIF	recurrent implantation iailure
G-CSF	granulocyte colony-stimulating factor (G-CSF)
IL-6	Interleukin-6
TLR	Toll-like receptor
TNFa	Tumor Necrosis Factor a
FET	frozen embryo transfer
MCP-1	monocyte chemoattractant protein-1
ICSI	Intracytoplasmic Sperm Injection
